# Factors contributing to low uptake of vaccination in underserved communities: a mixed-method diagnostic design in Southwest Region of Cameroon

**DOI:** 10.11604/pamj.2026.53.123.49613

**Published:** 2026-03-11

**Authors:** Edgar Mandeng Ma Linwa, Pamela Oben Besong, Nelson Sontsa Njedock, Jamin Ghangha, Ismail Shifu, Samuel Nkengfua, Martha Ngoe, Michael Ngenge Budzi, Neris Mabou Nfor, Kibu Odette Dzemo, Dickson-Shey Nsagha

**Affiliations:** 1 Rural Doctors, Buea, Cameroon,; 2 Regional Delegation for Public Health, Southwest, Buea, Cameroon,; 3 Faculty of Health Sciences, University of Buea, Buea, Cameroon

**Keywords:** Zero-dose, vaccine hesitancy, Southwest Cameroon, mixed-methods, convergent parallel design

## Abstract

**Introduction:**

in Cameroon, the Southwest Region (SWR) has a high zero-dose (ZD) prevalence (31% in 2021), undermining global immunization goals. This region also experiences sociopolitical unrest, which contributes to low vaccination uptake in underserved communities. This study aimed to quantify the prevalence, identify associated factors, explore barriers and facilitators, and elucidate socio-cultural drivers of zero-dose.

**Methods:**

a convergent parallel mixed-method design integrated a cross-sectional survey (parents of children <2 years) and two focused group discussions (FGDs, n=17) in Tombel and Eyumojock, two health districts with the lowest DTP3 coverage in the SWR. Multistage sampling targeted six community types: remote, conflict-affected, religious, displaced, urban slums, and ethnic minorities. Surveys, based on WHO Demographic and Health Survey modules and the Health Belief Model, assessed zero-dose status, demographics, and vaccine acceptance. Focused group discussions probed barriers, beliefs, and influencers. Quantitative data were analysed using univariable and multivariable logistic regression while qualitative data underwent thematic coding (NVivo 12). Meta-inferences combined findings to inform future interventions.

**Results:**

the prevalence of ZD children was 9.2% (39/424). Despite this, vaccine willingness was high (98.1% overall; 89.7% among parents of ZD children). Multivariable analysis identified disagreement that vaccines are a major advancement for humanity (aOR = 2.35, 95% CI: 1.08-5.10; p= 0.031) and lower self-efficacy/confidence in ensuring vaccination (aOR = 0.50, 95% CI: 0.25-1.00; p= 0.049 for high confidence) as significant predictors of ZD status. Perceiving diseases as not serious was marginally associated (aOR = 1.86, 95% CI: 0.93-3.72; p= 0.079). Qualitative themes highlighted access barriers (distance, stockouts, insecurity), misinformation (e.g., population control myths), and fears of poor administration. Trusted community leaders were key facilitators.

**Conclusion:**

the primary barrier to vaccination in this conflict-affected region is not willingness but access and logistical efficacy, compounded by misinformation. High acceptance rates provide a foundation for interventions. Tailored strategies must prioritize mobile outreach, supply chain resilience, and deploying trusted community leaders for education to reduce ZD prevalence and advance the Immunization Agenda 2030.

## Introduction

In 2024, World Health Organization (WHO) and United Nations International Children's Emergency Fund (UNICEF) reported that 89% of infants globally (115 million) received at least one diphtheria, tetanus and pertussis (DTP) vaccine dose and 85% (109 million) completed all three, representing gains of 171,000 and 1 million children compared to 2023 [1]. Yet, 20 million missed at least one DTP vaccine dose, including 14.3 million zero-dose children, which is 4 million above the 2024 Immunization Agenda 2030 target and 1.4 million more than in 2019, highlighting the urgent need to address vaccine hesitancy and access barriers in underserved communities [1]. In Cameroon, full vaccination coverage for children aged 12-23 months grew from 40% in 1991 to 52% in 2018 but fell 1% from 2011, with ZD children doubling from 5% to 10% in that period, while WHO/UNICEF National Immunization Coverage (WUENIC) data show DTP1 coverage remained stable at 75% and DTP3 coverage slightly increased from 67% in 2019 to 68% in 2022, despite COVID-19-related urban declines [2,3]. In Yaoundé (Cameroon), 37% of children aged 12-59 months were fully immunized, while 16% had never received any vaccine; living in wealthier households, having a mother with primary or higher education, and living with either parent significantly reduced the likelihood of being zero-dose [4]. In 2021, the highest rates of ZD children were reported in the Southwest (31% of children) and Northwest (24%) regions of Cameroon, two regions affected by sociopolitical unrest [3]. It is likely that the burden and factors linked to vaccine hesitancy and zero-dose vaccination in rural, conflict-affected areas differ markedly from those in stable regions like Yaoundé. For example, in Ethiopia, being unmarried [Odds Ratio (OR)=1.8], older child age (24-35 months) (OR=3.7), and greater distance to health facilities (OR=1.4), were additional factors linked to ZD prevalence [5]. Other factors fuelling ZD prevalence in sub-Saharan Africa include no formal paternal education, limited decision-making power in mothers, financial barriers, lack of media access, low community literacy and reduced country-level health expenditure [6]. Limited data on ZD and vaccine hesitancy drivers in conflict zones like Cameroon's Southwest region highlight the need for mixed-method research to understand complex phenomena and inform health policymakers, aligning with Newman *et al*. research purposes of understanding phenomena and informing constituencies [7]. The objectives are threefold: i) describe ZD prevalence and correlates; ii) explore barriers and facilitators to vaccination; and iii) explain relationships between socio-cultural factors and hesitancy.

## Methods

This study adhered to the guidelines for Conducting and Reporting Mixed Research in the Field of Counselling and Beyond [8]

**Study design:** the study utilised a fully mixed concurrent equal-status convergent parallel design collecting and analysing quantitative (cross-sectional survey) and qualitative (FGDs) data simultaneously during the same phase, with equal emphasis to address the research questions [9]. This design will enable triangulation of quantitative statistical associations (e.g., prevalence) with qualitative narratives (e.g. barriers) for corroboration, validation, and a comprehensive understanding of low uptake in low-performing settings. The convergent design was chosen due to limited data collection time, equal value of both data types, and the research team´s expertise in quantitative and qualitative methods. A pragmatic paradigm guided the study, integrating diverse data under a unified framework focusing on practical solutions rather than absolute truths, judging truth by its consequences, and supporting mixed methods despite philosophical inconsistencies [10].

**Research questions:** the study was guided by mixed research questions: i) What is the prevalence of ZD children in the Southwest region of Cameroon, and what demographic and access factors are associated? ii) What are parents´ perceptions and experiences of vaccination barriers? iii) How do quantitative associations align with qualitative barriers (e.g., trust, myths) to explain low uptake?

### Sampling procedures

**Quantitative phase:** the quantitative phase employed multistage sampling. First, a purposive selection of the two health districts (Tombel and Eyumojock) with the lowest reported DTP3 coverage in the Southwest Region (39% and 35%, respectively) was made based on unpublished 2023 EPI data. Within these districts, health areas and communities were purposively selected to represent six predefined community types: remote/rural, conflict-affected, religious groups, displaced populations, urban slums, and ethnic minorities. The sample size for the cross-sectional survey was calculated to provide a precise estimate of the zero-dose (ZD) prevalence, the study´s primary quantitative outcome. Based on the most recent available regional estimate of 31% ZD prevalence in the Southwest Region in 2021 [3], a minimum sample size was determined using the single proportion formula:

$$n=\frac{\left(Z^{2}\times P\left(1-P\right)\right)}{d^{2}}$$

Where Z = 1.96 (95% confidence level), P = 0.31 (expected proportion), and d = 0.05 (margin of error). This calculation yielded a minimum sample of 328 participants. Accounting for a design effect of 1.2 due to the multistage sampling and anticipating a potential non-response rate of 10%, the final target sample size was inflated to 432 participants. Furthermore, to ensure sufficient statistical power for the planned multivariable logistic regression analysis, we applied the rule of thumb requiring a minimum of 10 events per predictor variable (EPV). With the least frequent outcome being ZD status, and an expected prevalence of 31% in a sample of 432, we anticipated approximately 134 ZD children. This far exceeds the requirement for stable estimation with up to 10 predictor variables, ensuring robust model performance

**Qualitative phase:** the qualitative phase employed purposive criterion-based sampling to engage community stakeholders such as parents, community health workers, and community leaders. Two FGDs (8-10 per group) were held, one in Eyumojock and one in Tombel, ensuring gender balance and community type representativeness, to generate information-rich perspectives, aligning with recommendations [11,12]. FGDs were chosen over individual interviews for their cultural fit in communal settings, fostering authentic dialogue and group synergy that reveal social norms and collective barriers [11,13]. Thematic analysis followed the process outlined by Braun and Clarke (2006) [14]. Limitations, such as potential group thinking and silent voices, were addressed through skilled moderation [12]. Findings are presented narratively with illustrative quotes; frequencies are not reported for FGD data to avoid inappropriate quantification of perspectives from a small, purposive sample. Full questionnaire and interview guide can be found in supplementary files. These sampling schemes ensured analytic generalisations and case-to-case transfer, rather than mere statistical representativeness [15].

**Data collection:** mixed data collection strategies included structured surveys and semi-structured FGDs [16]. Questionnaires were adapted from a WHO-standard demographic and health survey module [17], and grounded in the Health Belief Model (HBM) [18]. The sample questionnaire used can be found in Annex 1. It assessed ZD vaccination status, demographics (age, education, income, ethnicity, religion, distance to health facility), household composition, knowledge (3 items), attitudes (4 items), perceived susceptibility/severity (2 items), benefits/barriers (4 items), and cues to action/self-efficacy (2 items). We also used the vaccine acceptance scale (20-item validated scale, α=0.97 historical [19], 0.47 current study). The questionnaires were administered via Kobo Toolbox by 15 trained enumerators in community settings, achieving a 100% completion rate. Focused group discussions used a piloted guide probing vaccination experience, community beliefs, influencers, and barriers. They were conducted in pidgin English (60 minutes, audio-recorded) in community halls. Visual aids (flipcharts) and skilled moderation ensured engagement. Quality was ensured through enumerator training, pilot testing, and audio verification by an independent researcher.

**Data analysis:** data analysis integrated the convergent parallel design and followed Onwuegbuzie and Teddlie´s (2003) seven-stage mixed-methods framework, which includes: a) data reduction; quantitative (descriptives, Chi-square for sociodemographics, HBM, vaccine acceptance; univariable and multivariable logistic regression logistic regression, R v4.2, odds ratio) and qualitative (thematic coding of FGD beliefs, barriers, NVivo 12); b) data display (tables, matrices); c) data transformation (quantitating themes e.g., myth frequency); d) data correlation (e.g., acceptance scores vs. themes); e) data consolidation (joint displays); f) data comparison (predictors vs. themes); g) data integration (meta inferences linking quantitative predictors and qualitative barriers to inform tailored interventions). Univariable logistic regression was performed for all potential predictors. Variables with p < 0.200 in univariable analysis or theoretical importance were considered for the initial multivariable model. A backward stepwise selection procedure was used. Model performance was assessed using the likelihood ratio test, classification accuracy, and pseudo-R^2^ measures. Variables such as hearing and visual impairment were excluded due to concerns about measurement validity. Backward stepwise selection was applied, iteratively removing non-significant variables until the best-fitting model was achieved. Zero-dose status was assessed via VS13 ('Has any of your children aged less than 2 years received any type of vaccination?’).

**Data validation and trustworthiness:** quantitative validity was ensured by addressing internal threats such as selection bias via multistage sampling across the six most vulnerable community types. External validity was also ensured through context-specific generalizability to conflict-affected settings. A key limitation was the low internal consistency (Cronbach´s α =0.47) of the multi-item vaccine acceptance scale, indicating poor reliability. Consequently, this scale was not used as a composite measure, and findings related to "vaccine acceptance" are based on the single global willingness item (VS14) [9,20]. Qualitative trustworthiness was achieved through thick descriptions of vaccination experiences, beliefs, and barriers using NVivo 12. Mixed-methods legitimation included: a) sample integration (parallel quantitative and qualitative samples targeting sociodemographics, HBM constructs, and community perspectives); b) weakness minimization (triangulation of survey predictors with FGD themes); and c) multiple validities (quantitative statistical rigor, qualitative credibility) [21]. Recall bias was mitigated through enumerator training and audio verification. Mixed criteria ensured design suitability, implementation fidelity, and integrative efficacy for zero-dose prevalence and barriers [22].

**Operational definition of terms:** i) zero-dose: children under two years who received no routine vaccinations, as reported by parents in survey item VS13 (*Has any of your children aged less than 2 years received any type of vaccination?*); ii) vaccine acceptance: parental willingness to vaccinate their child under two for some or all routine vaccines, measured by survey item VS14 (*Would you consider getting your child vaccinated against common vaccine-preventable diseases like diphtheria, tetanus, pertussis- hepatitis B and Haemophilus influenzae type b*?”); ii) self-efficacy/confidence: parents´ belief in their ability to ensure their child´s vaccination, assessed by survey item SS15 through a 6 point Likert scale; iv) remote community: areas with limited health facility access (e.g., >30-minute walk), identified by geographic isolation in Tombel and Eyumojock; v) conflict-affected community: areas impacted by ongoing violence or insecurity, that have disrupted local health services and mobility; vi) religious community: groups defined by shared religious beliefs influencing health decisions, and uncategorisable in other community types; vii) displaced community: populations forced to relocate due to conflict or other crises, per parental self-report in the survey; viii) urban slums: informal urban settlements with poor infrastructure and limited health access, identified by residential characteristics in the survey; ix) ethnic minorities: groups with distinct cultural or linguistic identities, per parental self-report in the survey.

**Ethical considerations:** the study received approval from the Institutional Review Board of the Faculty of Health Sciences (N° 2024/2572-08/UB/SG/IRB/FHS of 28 August 2024). Informed consent was obtained, confidentiality ensured, and no incentives were provided. The report adheres to validated reporting guidelines [8].

## Results

**Quantitative analysis:** a total of 424 parents were surveyed. The prevalence of zero-dose vaccination was 9.2% (39/424) and did not differ significantly across community types (χ^2^ 4.59, p=0.468). Strikingly, stated willingness to vaccinate was very high overall (98.1%) and among parents of ZD children (89.7%). The final multivariable logistic regression model (n=423) included three predictors (Table 1). The model fit was significant (Likelihood Ratio χ^2^=14.40, df=3, p=0.002) but explained limited variance (Nagelkerke R^2^=0.07). The significant predictors were: i) lower self-efficacy/confidence in ensuring child vaccination (protective effect of high confidence: aOR = 0.50, 95% CI: 0.25-1.00; p = 0.049); ii) disagreement that vaccines are a major advancement for humanity (aOR = 2.35, 95% CI: 1.08-5.10; p = 0.031); iii) perceived seriousness of vaccine-preventable diseases was marginally associated (aOR = 1.86, 95% CI: 0.93-3.72; p = 0.079).

**Table 1:** factors associated with zero-dose vaccination status (n=424)

Variable	Factor category (reference in parentheses)	Prevalence of factor category in zero-dose children n=39	Prevalence of factor category in vaccinated children n=379	Unadjusted OR (95% CI)	P-value	Adjusted OR (95% CI)	P-value
Respondent age (n=418)	≥35 years (vs 21-34 years)	22 (56.41%)	174 (45.91%)	1.56 (0.80-3.03)	0.188	_	_
Relationship with child (n=418)	Father / Other (vs Mother)	5 (13.16%)	29 (7.63%)	1.80 (0.65-4.95)	0.255	_	_
Marital status (n=420)	Single/Divorced/Widow (vs Married/cohabitating)	22 (56.41%)	154 (40.42%)	1.93 (0.99-3.76)	0.052	_	_
Hearing impairment (n=419)	Yes (vs No/Not sure)	20 (52.63%)	94 (24.67%)	3.25 (1.66-6.34)	0.001	_	_
Visual impairment (n= 411)	Yes (vs No/Not sure)	16 (43.24%)	75 (20.05%)	2.87 (1.44-5.69)	0.003	_	_
Willingness to vaccinate child (n=422)	No / Not sure (vs Yes-some or all)	4 (10.26%)	10 (2.61%)	4.27 (1.27-14.34)	0.019	_	_
Very/extremely worried child will contract VPDs (n=422)	No (vs Yes)	24 (61.54%)	293 (76.5%)	0.50 (0.25-0.99)	0.046	_	_
Believes vaccines protect child from diseases (n=423)	Disagree/Strongly disagree/Neutral/Don’t know (vs Agree/Strongly agree)	7 (17.95%)	34 (8.85%)	2.25 (0.92-5.49)	0.074	_	_
Perceived seriousness of vaccine-preventable diseases (n=420)	Not serious at all / Slightly / Neither (vs Very/Extremely serious)	19 (50.0%)	111 (29.06%)	2.34 (1.20-4.55)	0.012	1.86 (0.93 - 3.72)	0.079
Cost perceived as likely/very likely barrier (n=415)	Yes (vs No)	15 (38.46%)	119 (31.65%)	1.39 (0.70-2.75)	0.341	_	_
Religion perceived as unlikely/very unlikely to accept vaccines	Yes (unlikely/very unlikely) (vs likely/very likely)	2 (5.13%)	61 (16.27%)	0.29 (0.07-1.22)	0.091	_	_
Self-efficacy: confidence to ensure child is vaccinated	Very/extremely confident (vs lower confidence)	17 (43.59%)	247 (65.34%)	0.43 (0.22-0.83)	0.013	0.50 (0.25-1.00)	0.049
Vaccines are a major advancement for humanity	Moderately Disagree/Strongly disagree (vs Agree/Strongly agree)	11 (28.95%)	50 (13.19%)	2.62 (1.23-5.60)	0.013	2.35 (1.08 - 5.10)	0.031

**Qualitative analysis:** two focus group discussions (FGDs) with parents in Eyumojock (n=9) and Tombel (n=8), identified barriers and facilitators to vaccination through the lens of the Health Belief Model (HBM). In Eyumojock, participants perceived high susceptibility to and severity of vaccine-preventable diseases, valuing vaccines for protection (EHA1, perceived benefits). However, some participants reported mixed feelings, with fear of injection pain reducing perceived benefits (EHA4, perceived barriers). Misinformation, such as vaccines causing population control (EHA5), heightened perceived barriers, alongside access issues like insecurity and stockouts (EHA3, EHA6). Community chiefs and religious leaders were key cues to action (EHA3, EHA4), with many participants suggesting incentives and education to enhance self-efficacy (EHA1, EHA4). In Tombel, participants endorsed vaccine efficacy (THA06, perceived benefits), but some feared poor administration (THA03, perceived barriers), and noted Apostolic Faith resistance (THA03, perceived barriers). Misinformation about vaccine harm was prevalent (THA02), undermining perceived benefits. Health workers and chiefs were trusted cues to action (THA03, THA04), with some participants recommending training and formal communication to boost self-efficacy (THA04, THA06). Across districts, misinformation and access barriers dominated, with Tombel emphasizing administration fears and Eyumojock highlighting insecurity. Full thematic analyses with thick descriptions for Eyumojock and Tombel can be found in Annex 1.

**Meta-inferences:** Table 2 presents the joint display of key meta-inferences and indicate: i) convergence on an access-acceptance paradox: high quantitative willingness (98%) converges with qualitative recognition of vaccine benefits, yet qualitative access barriers explain the quantitative link between low self-efficacy and ZD status; ii) convergence on misinformation: quantitative disagreement with vaccines as an advancement aligns with qualitative myths, reducing perceived benefit. iii) divergence on severity: qualitative discussions acknowledged disease severity, but this did not emerge as a strong differentiating factor for ZD status in the quantitative model, suggesting other factors are more salient.

**Table 2:** meta-inferred joint display of qualitative and quantitative data

Meta-inference (integrated finding)	Supporting quantitative evidence	Supporting qualitative evidence	Implications for intervention
1. High vaccine willingness exists alongside major access barriers, creating a critical implementation gap.	Overall vaccine willingness: 98.1%. Willingness among parents of zero-dose children: 89.7%.	Dominant themes of stockouts ("if the mami them cam then vaccine no be available"), long distances, transportation costs, and insecurity ("Amba di always parade those areas").	Shift focus from demand creation to reliable service delivery. Prioritize mobile clinics, supply chain resilience, and flexible scheduling to overcome physical and security barriers.
2. Low parental confidence (self-efficacy) is a key risk factor, driven by logistical obstacles, not just attitude.	Lower self-efficacy/confidence significantly associated with zero-dose status (aOR=0.50 for high confidence).	Parents expressed feeling unable to vaccinate due to unpredictable vaccine availability, cost of travel, and clinic closures, undermining their perceived ability to act.	Boost self-efficacy by making vaccination predictable and easy. Implement community-based delivery, fixed advance schedules, and remove point-of-care costs.
3. Misinformation undermines the perceived benefit and value of vaccination.	Disagreement that "vaccines are a major advancement for humanity" strongly associated with zero-dose status (aOR=2.35).	Prevalent narratives of vaccines as tools for "population control," "infertility," or being "fake" (e.g., "government want eliminate male children").	Counter misinformation by co-opting trusted community channels. Engage chiefs, pastors, and local health workers as credible messengers for benefit-focused education.
4. Community leaders are pivotal, trusted facilitators for health decisions.	Not a direct quantitative measure, but aligns with high willingness influenced by external cues.	Consistent identification of chiefs, quarter heads, and religious leaders as the most influential figures for mobilizing compliance and building trust.	Formally integrate and train community leaders as vaccine advocates. Empower them to lead mobilization, disseminate correct information, and legitimize outreach efforts.
5. Perceived severity of diseases is a weaker motivator compared to tangible access and benefit perceptions.	Perceived low disease seriousness was only marginally associated with zero-dose status (p=0.079).	While disease severity was acknowledged, discussions focused overwhelmingly on immediate practical barriers and fears about vaccine safety/intent.	Emphasize practical benefits and safety in messaging. Move beyond fear-based communication about diseases to highlight vaccination as a feasible, positive community norm.

## Discussion

This mixed-methods study in Cameroon´s conflict-affected SWR reveals a critical paradox: extremely high parental willingness to vaccinate (98.1%) coexists with a persistent, though lower-than-expected, zero-dose prevalence of 9.2%. This finding challenges the framing of the issue purely as "vaccine hesitancy" and redirects focus to structural and delivery system failures as the primary drivers of zero-dose status in this context. The significantly lower ZD prevalence found (9.2% vs. the 31% regional estimate for 2021) requires careful interpretation. It may reflect genuine progress due to intensified local microplanning and outreach campaigns in these districts [3,23]. However, methodological factors, including potential sampling bias towards more accessible households within insecure areas and the reliance on unverified parental recall for ZD status, may also contribute to an underestimate. The true prevalence likely lies between these figures, but the dramatic difference underscores the potential impact of focused community engagement. Our regression model, while identifying significant cognitive predictors (low perceived benefit, low self-efficacy), had critical limitations. Its inability to correctly identify any ZD child (0% sensitivity) strongly illustrates that, unmeasured, predominantly structural variables, such as stockouts, episodic insecurity, and variable service delivery, may be the principal determinants. This aligns perfectly with our qualitative findings where these access barriers dominated narratives. The quantitative link between low self-efficacy and ZD status likely captures parents' realistic assessment of these systemic obstacles, not a psychological deficit. The high willingness to vaccinate, even among most ZD parents, is a crucial asset. It suggests demand is not the bottleneck. This aligns with studies emphasizing systemic barriers in fragile settings [24]. and contrasts with studies from more stable urban areas where hesitancy is more prominent [4]. Our qualitative data on trusted leaders (chiefs, pastors) as key influencers corroborates evidence from Ethiopia and elsewhere on the importance of community networks [5].

The 9.2% zero-dose prevalence aligns with WHO/UNICEF national estimates for Cameroon (10% in 2018), but is notably lower than the 31% reported for the Southwest region in 2021, likely reflecting localized interventions such as microplanning (including district-level mapping and community engagement to target zero-dose children), the Handshake Model integrating vaccination with community services, and geospatial catch-up campaigns targeting missed communities [3,23]. High vaccine acceptance suggests awareness of vaccine benefits, corroborated by qualitative themes valuing disease prevention. However, misinformation, particularly myths about population control, undermines uptake, though agreement with vaccine protection indicates that most ZDV parents believe in vaccine efficacy, suggesting potential for targeted education. Low self-confidence may stem from logistical barriers (e.g., stockouts, insecurity in Eyumojock), reducing parents´ perceived ability to vaccinate. Tombel´s focus on administration fears reflects distrust in health worker competence, a concern amplified in conflict zones with strained health systems [24,25]. Eyumojock´s emphasis on insecurity highlights how sociopolitical unrest disrupts access [24].

Limitations include the cross-sectional design, preventing causal inference; the poor reliability of the vaccine acceptance scale, confining acceptance analysis to a single item; potential social desirability bias in stated willingness; and possible under-sampling of the most inaccessible, security-compromised households, potentially biasing the ZD prevalence estimate downwards. The use of parent-reported, unverified vaccination status is a notable source of potential misclassification. For policy and practice, the primary implication is to shift resources from persuasive communication campaigns, which our data suggest are less urgently needed, toward solving logistical and access problems. Investments should prioritize: i) predictable, resilient vaccine supply chains to eliminate stockouts; ii) reaching the last mile through funded mobile clinics and community health worker networks; formally engaging and training trusted community leaders as vaccine advocates to counter misinformation and mobilize communities. Future research should employ longitudinal designs and integrate verified vaccination data with real-time metrics of access (e.g., stockout frequency, security incidents) to better model the dynamics of zero-dose in conflict settings.

## Conclusion

In conflict-affected Southwest Cameroon, the barrier to childhood vaccination may not be parental refusal but systemic failure. High willingness to vaccinate may be hindered by stockouts, distance, insecurity, and misinformation. This demands a strategic shift from persuasion to access: prioritize reliable supply chains, mobile outreach to remote communities, and formal engagement of trusted local leaders to counter myths. These actions are critical to convert high acceptance into coverage and achieve immunization equity.

### What is known about this topic

- Global vaccination coverage has improved, with 89% of infants receiving at least one DTP dose in 2024, yet 14.3 million zero-dose children remain, particularly in conflict-affected and underserved regions (WHO/UNICEF, 2025);
- In Cameroon, full vaccination coverage is low (52% in 2018), with zero-dose rates higher in conflict zones like the Southwest (31% in 2021);
- Although it is likely that the burden and factors linked to vaccine hesitancy and zero-dose prevalence in conflict-affected areas differ markedly from those in stable regions, comprehensive data in literature is lacking.

### What this study adds

- Prevalence of zero-dose in Southwest Cameroon was 9.2%, highlighting progress despite conflict, with high vaccine acceptance (98.1%) even among parents of zero-dose children;
- Specific barriers (misinformation, stockouts, administration fears) and associated factors (perceived benefits, self-efficacy barriers, disagreement with vaccine as an advancement), were identified through a mixed-method approach;
- Interventions such as mobile clinics, community education, and engagement of trusted leaders, tailored to conflict-affected settings, may be a model for addressing zero-dose challenges in alignment with Immunization Agenda 2030 goals.

## Annexes

**Annex 1**: supplementary materials (PDF - 440KB)
